# Spectrum of Kidney Disorders Associated with T-Cell Immunoclones

**DOI:** 10.3390/jcm11030604

**Published:** 2022-01-25

**Authors:** Alexis Piedrafita, François Vergez, Julie Belliere, Nais Prades, Magali Colombat, Antoine Huart, Jean-Baptiste Rieu, Stéphanie Lagarde, Arnaud Del Bello, Nassim Kamar, Dominique Chauveau, Camille Laurent, Lucie Oberic, Loïc Ysebaert, David Ribes, Stanislas Faguer

**Affiliations:** 1 Centre de Référence des Maladies Rénales Rares, Département de Néphrologie et Transplantation d’Organes, Centre Hospitalier Universitaire de Toulouse, F-31000 Toulouse, France; piedrafita.a@chu-toulouse.fr (A.P.); belliere.j@chu-toulouse.fr (J.B.); huart.a@chu-toulouse.Fr (A.H.); delbello.a@chu-toulouse.fr (A.D.B.); kamar.n@chu-toulouse.fr (N.K.); chauveau.d@chu-toulouse.fr (D.C.); ribes.d@chu-toulouse.fr (D.R.); 2 UMR 1297 (Institut des Maladies Métaboliques et Cardiovasculaires-Team 12), Institut National de la Santé et de la Recherche Médicale, F-31000 Toulouse, France; 3 Laboratoire d’Hématologie, Institut Universitaire du Cancer de Toulouse-Oncopôle, Centre Hospitalier Universitaire de Toulouse, F-31000 Toulouse, France; vergez.Francois@iuct-oncopole.fr (F.V.); prades.nais@iuct-oncopole.fr (N.P.); rieu.jean-baptiste@iuct-oncopole.fr (J.-B.R.); lagarde.stephanie@iuct-oncopole.fr (S.L.); 4 Faculté de Médecine Rangueil, Université Paul Sabatier-Toulouse III, F-31000 Toulouse, France; colombat.magali@iuct-oncopole.fr (M.C.); laurent.camille@iuct-oncopole.fr (C.L.); ysebaert.loic@iuct-oncopole.fr (L.Y.); 5 Département d’Anatomopathologie, Institut Universitaire du Cancer de Toulouse, Centre Hospitalier Universitaire de Toulouse, F-31000 Toulouse, France; oberic.lucie@iuct-oncopole.fr; 6 Service d’Hématologie, Institut Universitaire du Cancer de Toulouse-Oncopôle, Centre Hospitalier Universitaire de Toulouse, F-31000 Toulouse, France

**Keywords:** immunoclones, large granular lymphocytic leukemia, renal fibrosis, T-cell receptor, *STAT3*, autoimmune disorders

## Abstract

Large granular T-cell leukemia is a clonal hematological condition often associated with autoimmune disorders. Whether small-sized T-cell clones that are otherwise asymptomatic can promote immune kidney disorders remains elusive. In this monocentric retrospective cohort in a tertiary referral center in France, we reviewed characteristics of 29 patients with T-cell clone proliferation and autoimmune kidney disorders. Next-generation sequencing of the T-cell receptor of circulating T-cells was performed in a subset of patients. The T-cell clones were detected owing to systematic screening (mean count 0.32 × 10^9^/L, range 0.13–3.7). Strikingly, a common phenotype of acute interstitial nephropathy was observed in 22 patients (median estimated glomerular filtration rate at presentation of 22 mL/min/1.73 m^2^ (range 0–56)). Kidney biopsies showed polymorphic inflammatory cell infiltration (predominantly CD3^+^ T-cells, most of them demonstrating positive phospho-*STAT3* staining) and non-necrotic granuloma in six cases. Immune-mediated glomerulopathy only or in combination with acute interstitial nephropathy was identified in eight patients. Next-generation sequencing (*n* = 13) identified a major T-cell clone representing more than 1% of the T-cell population in all but two patients. None had a mutation of *STAT3*. Twenty patients (69%) had two or more extra-kidney autoimmune diseases. Acute interstitial nephropathies were controlled with corticosteroids, cyclosporin A, or tofacitinib. Thus, we showed that small-sized T-cell clones (i.e., without lymphocytosis) undetectable without specific screening are associated with various immune kidney disorders, including a previously unrecognized phenotype characterized by severe inflammatory kidney fibrosis and lymphocytic JAK/STAT activation.

## 1. Introduction

The development of new molecular diagnosis tools, including multicolor flow cytometry, polymerase chain reaction of the T-cell receptor (TCR), and next-generation sequencing, has provided new opportunities to refine the phenotypes and molecular mechanisms of immune disorders of unknown origin. This approach helped to relate large granular lymphocytic leukemia (LGL), a condition frequently associated with autoimmune diseases, with a clonal or oligoclonal proliferation of cytotoxic T-cells [1].

LGL is considered a reactive process with extreme clonal skewing [1,2]. Most LGL cells harbored a CD3^+^ CD8^+^ phenotype, and more rarely expressed the CD4 marker, or had a natural killer (NK) phenotype [1]. Activation of the JAK/STAT pathway in CD8^+^ T-cells is observed in most if not all LGL clones and is driven by acquired mutations of the *STAT3* or very rarely *STAT5b* genes in 5% to 30% of patients [3,4,5]. *STAT3* mutations are considered a secondary event during clonal expansion, but LGL can evolve and progress because of persistent antigenic drive, even if *STAT3* is not mutated [4].

Cytopenia (autoimmune hemolytic anemia and neutropenia) and rheumatoid arthritis are the most frequent immune disorders associated with LGL, but a wide panel of autoimmune diseases were also observed in patients with LGL, including hematological (red cell aplasia, pernicious anemia, thrombocytopenia), neurological (myasthenia gravis, polyradiculoneuropathy), or rheumatic (Sjögren’s syndrome, lupus) disorders [1,2]. To date, only seven cases of renal disease related to LGL have been reported, including renal infiltration by the LGL or heterogeneous glomerulopathies (focal and segmental glomerulosclerosis, vasculitis with anti-glomerular basement membrane antibodies, heavy-chain amyloidosis, glomerulonephritis with endocapillary proliferation) [6,7,8,9,10,11,12]. Recently, we reported two cases of inflammatory renal fibrosis associated with a small-sized T-cell clone (i.e., a clonal population not associated with lymphocytosis and thus requiring dedicated immunophenotyping to be identified) [13]. We demonstrated that these immunoclones may promote phenotypic changes of the epithelial renal cells favoring renal fibrosis. In both cases, steroids’ responsiveness was dramatic. Bone marrow fibrosis induced by the LGL clone was also previously demonstrated [14], but whether such small-sized LGL proliferation may promote multi-organ inflammatory fibrosis remains elusive. Previous studies using multicolor flow cytometry also suggested that the size of the circulating LGL clone is highly heterogeneous and may vary from 0.1 to >10 × 10^9^/L, supporting the use of a new functional definition based on the need to (a) treat the immunologic effects of the immunoclones (T-cell immunoclones, TIC), (b) to treat the neoplastic effects of leukemia (LGL leukemia), or (c) to only monitor the patients with an asymptomatic circulating T-cell clone (asymptomatic patients, T-cell clone of undetermined significance (TCUS)) [15,16,17].

In this study, we aimed to describe the immune kidney disorders associated with T-cell immunoclones.

## 2. Methods

In this retrospective monocentric study, we included all adult patients with a T-cell immunoclone detectable in the blood or organ and followed at the department of Nephrology, Clinical Immunology, and Organ Transplantation of the University Hospital of Toulouse (France). The inclusion period was January 2010 to September 2020. The study was conducted in compliance with the Good Clinical Practice protocol and the Declaration of Helsinki principles, as well as French law regarding retrospective observational studies. According to the recommendations of the Institutional Review Board of the University Hospital of Toulouse, written informed consent was waived.

### 2.1. Patients’ Characteristics

Clinical and biological parameters were collected through a standardized screening of the patients’ hospital records. Routine kidney pathology consisted of light microscopy and immunostaining directed against IgG, IgA, IgM, kappa and lambda light chains, CD3, and CD20. The expression of phospho-*STAT3* was performed on 3 µm-thick sections of available formalin-fixed paraffin-embedded whole-tissue biopsies of the kidney, liver, or lymph node and tested using the Ventana Benchmark XT immunostainer (Ventana, Tucson, AZ, USA). Samples were then stained with the anti-phospho-*STAT3* antibody (dilution 1/100; RM261; Diagomics, Blagnac, France). Co-immunostaining could not be performed due to the small amount of tissue available, but all biopsies were reviewed by a pathologist (C.L.) to assess whether the cellular compartment p-*STAT3* was identified (endothelial, epithelial, lymphoid cells).

### 2.2. Immunological Analyses

Indication to screen for immunoclones was lymphocytosis (>5000/mm^3^), autoimmunity, or inflammatory fibrosis of unknown origin. Blood samples were collected in EDTA tubes and usually tested within 48 h of collection. The quantification of lymphocyte subpopulations was determined by flow cytometry using DuraClone IM phenotyping Basic Tubes and Flow Fluorospheres (Beckman Coulter, Brea, CA, USA). Briefly, the cells were stained with a panel of antibodies targeting CD3 (clone UCHT1), CD5 (clone L17F12), CD56 (clone NCAM16. 2), CD57 (clone HNK-1), HLA-DR (clone G46-6) (BD Biosciences, Franklin Lakes, NJ), CD16 (clone 3G8), CD8 (clone B9.11) (Beckman Coulter, Brea, CA, USA), and CD4 (clone RPA-T4) (Biolegend, San Jose, CA, USA). Flow cytometry data were acquired on a Navios instrument and analyses were performed with Kaluza software (Beckman Coulter, Brea, CA, USA).

Diagnosis of immunoclones relied on the identification of a circulating lymphocyte population with immune phenotype characteristics of LGLT, LGLNK, or CD4+ T-cell clones, or with a demonstration of organ infiltration by a T-cell clone. T-cell clonality was studied on DNA extracted from circulating cells or frozen tissue by multiplex PCR targeting the *TRB* and *TRG* genes, following DNA quality assessment by a control ladder PCR. Biomed-2 primers were used with standard PCR conditions. Amplification products were analyzed by electrophoresis on polyacrylamide gel.

### 2.3. STAT3 and TCR Sequencing

To estimate the size of the immunoclone population and search for *STAT3* acquired mutations, next-generation sequencing of the T-cell receptor (TCR) Vd and Jd complementarity-determining region 3 (CDR3) and the *STAT3* gene was performed with PCR amplicon-based libraries. The amplicons were generated by specific multiplexed PCRs, one for *STAT3* exon 20 and 21, and two for TCRγ and TCRβ rearrangements (primers based on the Biomed-2 European collaborative study [18]). The obtained PCR amplicons were then subjected to an indexing PCR with the Nextera XT Index kit V2 (Illumina, San Diego, CA, USA). The libraries were sequenced with a MiSeq sequencer (Illumina, San Diego, CA, USA) and Miseq Reagent kit V2 (paired-end sequencing 2 × 150 cycles). For *STAT3* mutations’ analysis, alignment was performed using the BWA aligner and variant calling was performed using FreeBayes and Mutect2 variant callers. For TCR rearrangements’ alignment and detection, we used the Vidjil web platform [19].

### 2.4. Statistical Analyses

Data were shown as medians and limits for continuous variables, and as numbers and percentages for discontinuous variables.

## 3. Results

### 3.1. Patients

**Figure 1:**
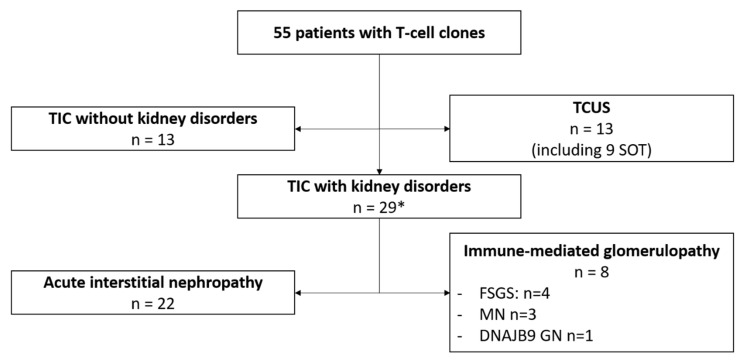
Flow-chart of the study. TIC, T-cell immunoclone; TCUS, T-cell clone of unknown significance; FSGS, focal and segmental glomerulosclerosis; MN, membranous nephropathy; GN, glomerulonephritis. * One patient had both acute interstitial nephropathy and immune-mediated glomerulopathy.

From January 2010 to November 2020, a chronic LGL population was identified in 55 patients, including 10 solid organ transplant recipients (accounting for 21% of the total peripheral blood lymphocyte counts (7; 51)) (Figure 1). The circulating clone was identified during the screening of asymptomatic lymphocytosis in 13 patients (24%, including nine solid organ transplant recipients), leading to the diagnosis of TCUS. The remaining 42 patients had TIC. In them, the T-cell clone was searched for to explore the mechanisms of polyautoimmunity or immune-mediated nephropathy. One kidney transplant recipient had recurrent episodes of acute interstitial nephropathy, formerly considered as acute cellular rejection, and myositis. No TCUS patient developed overt T-cell lymphoma, B symptoms, or splenomegaly, and none received anti-lymphoma therapy. Among the 13 TCUS cases, 11 were considered reactive LGL proliferation.

In this study, we addressed the characteristics of 29 out of the 42 TIC patients who developed kidney disease (male gender *n* = 14, 48%; median age at diagnosis 67 years, range 35–84) (Table 1). Twenty patients (49%) had two or more autoimmune diseases. Six patients had polyclonal hypergammaglobulinemia (≥15 g/L).

### 3.2. Kidney Presentation

Among the 29 patients, 22 had acute interstitial nephropathy (i.e., no hypertension, tubular proteinuria, urinary leukocytes, no urinary red blood cells, rapidly progressive renal failure, median estimated glomerular filtration rate at presentation of 22 mL/min/1.73 m^2^ (range 0–56)), with non-necrotic granuloma in 6 cases. One patient had both granulomatous interstitial nephropathy and IgG4^+^ PLA2R^-^ membranous nephropathy. When assessed, immunostaining showed polymorphic infiltration of inflammatory cells, with predominant CD3^+^ T-cell infiltration without overt CD4/CD8 skewing. Predominant CD3^+^ T-cell proliferation did not have the monomorphic pattern usually observed in T-cell lymphoma. In all except two patients, the introduction of a new drug in the last four weeks was ruled out. Of note, the diagnosis of hyper-IgG4 syndrome was ultimately retained in two cases and acute interstitial nephropathy occurred in three patients who previously received an immune checkpoint inhibitor.

Strikingly, a severe inflammatory fibrosis phenotype also involved the liver in three patients, a finding reminiscent of liver, pleura, node, bone marrow, and salivary gland involvement in patients without kidney involvement. Biopsies of extra-kidney involvements showed the same inflammatory pattern.

In eight patients, glomerulopathy was identified, including focal and segmental glomerulosclerosis (FSGS, *n* = 4), membranous nephropathy (*n* = 3), and DNAJB9^+^ fibrillar glomerulonephritis (*n* = 1). In one patient with nephrotic syndrome and FSGS, activated CD8^+^ CD56^+^ T-cells were shown within the glomerular capillary tuft.

**Figure 2:**
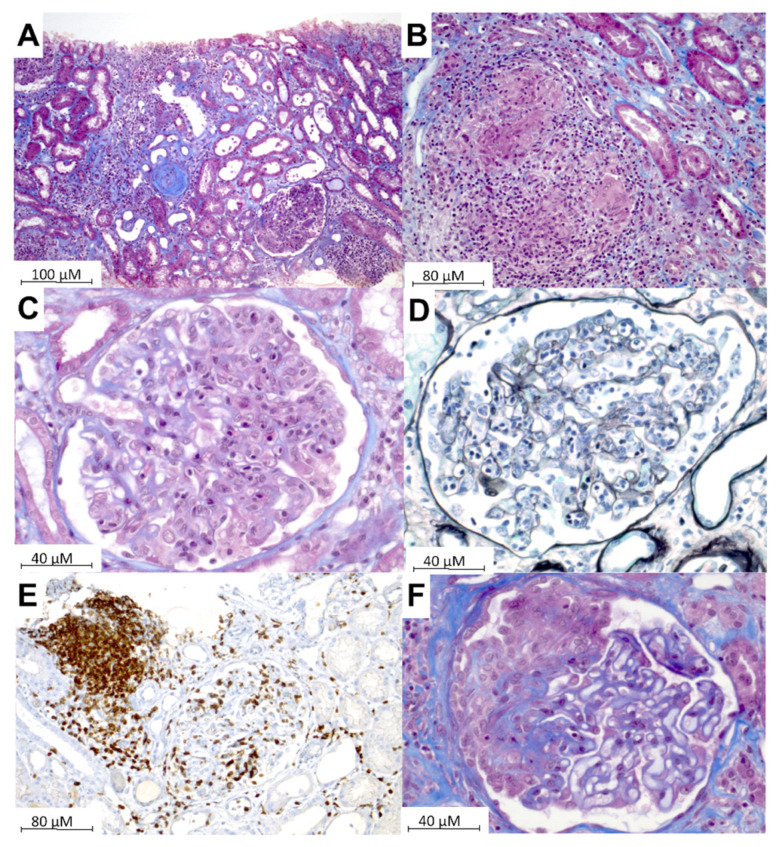
Kidney pathology. (**A**) Acute interstitial nephropathy with polymorphic infiltration of inflammatory cells and interstitial edema (Masson’s trichroma). (**B**) Interstitial granuloma (Masson’s trichroma). (**C**–**E**) Glomerulonephritis with CD3+ T-cells’ endocapillary proliferation ((**C**) Masson’s trichroma staining, (**D**) Jones’ staining, (**E**) CD3+ immunostaining in brown). (**F**) Membranous nephropathy with extra-capillary cell proliferation (Masson’s trichroma).

Figure 2 shows representative sections of the kidney biopsies performed in the patients included in our series.

### 3.3. Characteristics of the Immunoclones

Among the 29 patients, all had a normal lymphocyte count. Systematic screening with T-cell immunophenotyping detected the abnormal population (median count of LGL cells at diagnosis 0.32 × 10^9^/L, range 0.13–3.7). No mutation of *STAT3* was identified in the 13 tested patients. The median size of the major clonal population represented 3.7% of the T-cell population (0.4; 13.1), as assessed by next-generation sequencing of the *TCRG* gene, and represented more than 1% of the T-cell population in all except two patients.

### 3.4. Outcomes

Frontline therapy included corticosteroids in 23 patients. Three patients did not receive systemic treatment. During follow-up, several lines of an immunosuppressive regimen were used (cyclosporin A *n* = 8, rituximab *n* = 8, methotrexate *n* = 1, tofacitinib *n* = 2, TNFα inhibitor *n* = 1, leflunomide *n* = 1) but none were associated with complete elimination of the clone. The variety of the immunosuppressive regimen relied on the multiple autoimmune diseases that successively developed in the patients (for instance, rituximab for hyper-IgG4 syndrome or membranous nephropathy). Cyclosporin A was introduced in nine patients because of corticosteroids’ refractoriness, dependency, or adverse effects, and led to a dramatic kidney response in four. Cyclosporin was stopped in these four individuals because of the adverse events and/or a lack of efficacy. The JAK inhibitor tofacitinib was administered in some patients owing to its activity in refractory large granular lymphocytes leukemia [20].

### 3.5. Mechanisms of Inflammatory Fibrosis

**Figure 3:**
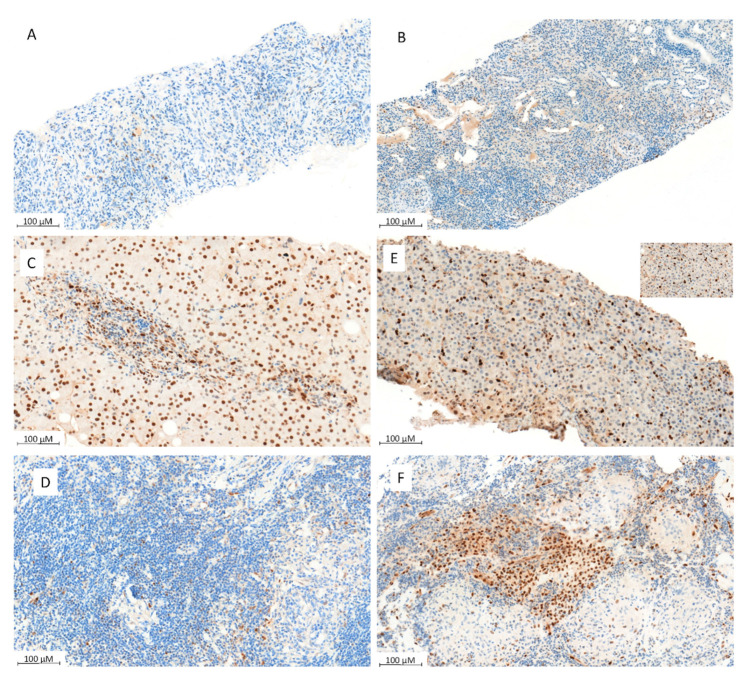
Phospho-*STAT3* immunostaining. (**A**,**B**) Representative cases of p*STAT3* staining in renal fibrosis showing nuclear expression in some lymphocytes and endothelial cells (**A** ×150 and **B** ×100). (**C**,**D**) Representative cases of p*STAT3* staining in a liver sample expressed by numerous lymphocytes and hepatocytes (**C** ×200) or highlighting the intra-sinusoidal T-LGL infiltrate (**D** ×150). (**E**,**F**) Representative cases of p*STAT3* staining in inflammatory fibrosis tissue (**E** ×100) and sarcoidosis-like lymph node (**F** ×150) showing nuclear expression mostly in reactive lymphocytes (**D**) and histiocytes (**F**) as well as in endothelial cells.

The identification of inflammatory fibrosis, sometimes accompanied by granuloma, prompted us to characterize the activity of the JAK/STAT pathway, a potential therapeutic target in both LGL and granulomatosis [20,21]. Phospho-*STAT3* immunostaining was performed on kidney samples from six patients, and in extra-kidney samples from seven additional patients (liver *n* = 5, lymph node *n* = 2), and showed various states of *STAT3* activation in lymphocytes in most biopsies (ranging from 1% to 80% of lymphocytes, Figure 3). In some patients, positive phospho-*STAT3* staining was also observed in endothelial or renal tubular cells.

In one patient with severe immune thrombocytopenia refractory to splenectomy, corticosteroids, rituximab, and thrombopoietin agonists, who developed vitiligo and kidney and node granulomatosis while receiving monthly injections of intravenous immunoglobulins, the kidney biopsy showed interstitial nephropathy with intense phospho-*STAT3* staining in lymphocytes and tubular cells (Figure 3). Administration of the oral JAK inhibitor tofacitinib was followed by dramatic improvement and led to complete remission of thrombocytopenia and vitiligo, but also of the hypermetabolic nodal target (as assessed by a 18F-FDG PET-CT scan), along with kidney function stabilization and immunoglobulins withdrawal.

## 4. Discussion

Despite major advances in the understanding of immune diseases, underlying biological mechanisms remain mostly elusive, precluding individualized and targeted therapy. Combining immunophenotyping, next-generation sequencing of the TCR CDR3 region, and pathology analyses, we showed that LGL proliferation and immunoclones are associated with various immune kidney disorders, including relapsing inflammatory kidney fibrosis with granulomatosis. The link between the LGL proliferation and the antibody-mediated glomerulopathy was more elusive and will require further studies. Additionally, how the detection of a circulating T-cell clone in a kidney transplant recipient may modify its follow-up and immunosuppressive treatment remains elusive. Our study suggests that, in a subset of patients, these clones are of clinical significance and might be targeted to avoid recurrent episodes of acute interstitial nephritis and to improve the long-term prognosis.

Owing to a high clonal proliferation index and a defect of apoptosis, LGL may lead to organomegaly, pancytopenia, and fatigue (LGL leukemia), but in an independent fashion, the production of inflammatory cytokines by LGL cells can promote autoimmunity and BM fibrosis [1]. This was recognized for a long time in patients with LGL leukemia and immune cytopenia or rheumatism. However, the role of small-sized LGL clones remained a matter of debate in patients with other immune disorders [15]. In our series, most patients had no lymphocytosis, and the size of the clonal population was below 0.5 × 10^9^/L in most patients, but the LGL population represented more than 1% of the circulating lymphocytes in almost all TIC patients with kidney involvement. Autoimmunity developed no matter the size of the clonal population. The development of several autoimmune diseases over several years in some patients, especially immune disorders previously associated with LGL [1], also supports the role of small-sized T-cell clones in autoimmunity or organ injury. This paradigm shift was already recognized in monoclonal B-cell lymphocytosis and plasma cell dyscrasia and led to the individualization of monoclonal gammopathy of renal significance and the development of new treatment algorithms [22]. Further studies will now have to refine the frequency of T-cell clones of renal significance and the clinical presentations that should prompt the search for LGL circulating cells.

One of the main findings of our study is the demonstration of LGL cells in several patients referred for interstitial nephropathy. Kidney pathology showed interstitial infiltration of inflammatory cells, especially CD3^+^ T-cells, sometimes accompanied by tubulitis and granuloma. Contrasting with kidney localization of T-cell lymphoma [23], infiltration with polymorphic immune cells suggestive of drug-induced nephropathy should be ruled out first. Sarcoidosis was formerly considered in some patients included in this series. Of note, the angiotensin-converting enzyme was not increased, and the outcome was favorable following cyclosporin A administration in four patients. The recent description of the activation of the JAK/STAT pathway in cutaneous T-cells of patients with refractory sarcoidosis [21] suggests common pathophysiological mechanisms and should prompt the search for LGL cells in atypical, frequently relapsing, or refractory sarcoidosis. It also points out how combined characterization of the circulating immune cell subpopulations and the activation state of tissue lymphocytes may help to tailor the immunosuppressive regimen of patients with complex autoimmune diseases. Hence, our study paves the way for an interventional study testing the efficacy and tolerance of JAK/STAT inhibition in patients with immune kidney disease and T-cell immunoclones [20]. Of note, because circulating T-cell clones may lead to organ injury with overt clonal infiltration (i.e., due to toxic soluble factors’ production or to modulation of other T-cell populations prone to induce autoimmunity), assessment of the JAK/STAT pathway activation is probably not the best way to identify patients who will respond to these drugs.

This study has some limitations, mainly related to its retrospective design. TCR sequencing was not available for all the patients included in the study and the evolution of the clone size could not be followed. Lastly, we could not isolate T-cell clones to address how they promote inflammatory organ fibrosis or which molecular pathways are involved. However, our findings now furnish a tremendous opportunity to better understand the immune–epithelial crosstalk within kidneys and identify new targetable fibrosis pathways.

## 5. Conclusions

We have shown that circulating T-cell clones that are otherwise asymptomatic can promote immune kidney disorders, including a phenotype characterized by severe inflammatory kidney fibrosis and lymphocytic JAK/STAT activation. We suggest performing dedicated flow cytometry of T-cells or searching for T-cell clonality within injured tissues in patients with polyautoimmunity and/or with acute interstitial nephropathy of unknown origin. Targeting the JAK/STAT pathway with the JAK inhibitor tofacitinib in this setting should be tested in a prospective trial.

## Figures and Tables

**Table 1:** Characteristics of the 29 patients with TIC and kidney involvement.

Pt	Age	Kidney Disease	Extra-Kidney Disorders	Clone Size	Treatment	eGFR at Last Follow-Up
1	70	Acute interstitial nephropathy		Tissue	Cst	CR
2	62	Acute interstitial nephropathy		23%	Cst, RTX	CR
3	55	Acute interstitial nephropathy	Thyroiditis, cholangitis	Tissue	Cst	ESKD
4	78	Acute interstitial nephropathy	Chronic myelomonocytic leukemia	16%	CsA	Stable
5	79	Acute interstitial nephropathy	Hepatitis, chronic neutropenia	27%	None	Early death
6	52	Acute interstitial nephropathy	Psoriasis	20%	None	Loss of follow-up
7	71	Acute interstitial nephropathy	Interstitial lung disease	31%	Cst	Steroid dependency
8	76	Acute interstitial nephropathy	Psoriatic arthritis, pleura infiltration	27%	Leflunomide, HCQ, MTX, anti-TNFa, Cst	Stable
9	49	Acute interstitial nephropathy		Tissue	Cst	ESKD
10	35	Acute interstitial nephropathy	Uveitis	11%	Cst, CsA	CR
11	52	Acute interstitial nephropathy	IgG4 syndrome	12%	Cst, RTX	Steroid dependency
12	48	Acute interstitial nephropathy		Tissue	Cst	CR
13	67	Acute interstitial nephropathy		15%	Cst	CR
14	81	Acute interstitial nephropathy		31%	Cst	CR
15	50	Acute interstitial nephropathy		14%	Cst, CsA	CR
16	66	Acute interstitial nephropathy	IgG4 syndrome	22%	Cst, RTX	CR
17	69	Acute interstitial nephropathy	Bullous pemphigoid, cholangitis	42%	Cst, CsA	CR
18	69	Acute interstitial nephropathy	Sjögren syndrome, myasthenia, interstitial lung disease	20%	Cst, RTX	Stable
19	56	Acute interstitial nephropathy	Myositis	14%	Cst, CsA	ESKD
20	46	Acute interstitial nephropathy	Uveitis, scleritis, thyroiditis	21%	Cst	CR
21	77	Acute interstitial nephropathy	Immune thrombocytopenia, vitiligo, interstitial lung disease	27%	Cst, RTX, IgIV, CsA, Tofacitinib	CR
22	84	Acute interstitial nephropathy	Anemia, thrombocytopenia	17%	CSt, CsA	No
23	75	Focal and segmental glomerulosclerosis		23%	CsA	CR
	72	Focal and segmental glomerulosclerosis		21%	Cst, CsA	CR
24	67	Focal and segmental glomerulosclerosis		22%	Cst, RTX, Tofacitinib	Steroid dependency
25	43	PLA2R^+^ membranous nephropathy	Immune thrombocytopenia	7%	RTX	CR
26	71	PLA2R^+^ membranous nephropathy	Polymyalgia rheumatica	14%	RTX	CR
27	76	PLA2R^+^ membranous nephropathy	Granulomatous hepatitis	12%	None	Loss of follow-up
28	58	Minimal change disease	Rheumatoid arthritis	25%	Cst, RTX, CsA	CR
29	44	DNAJB9^+^ fibrillary glomerulonephritis	Myositis	19%	None	Stable

Pt, patient; CR, complete response; Cst, corticosteroids; RTX, rituximab; CsA, ciclosporin-A; HCQ, hydroxychloroquine; MTX, methotrexate; TNFa, tumor necrosis factor-alpha; ESKD, end-stage kidney disease; IgIV, intravenous immunoglobulins; eGFR, estimated glomerular filtration rate.

## Data Availability

The data that support the findings of this study are available from S.F., but restrictions apply to the availability of these data, which were used under license for the current study, and so are not publicly available. Data are, however, available from the authors upon reasonable request and with permission from S.F.
